# Appendicitis Mimicking Urinoma: A Challenging Emergency Presentation Secondary to Ureteric Stone

**DOI:** 10.7759/cureus.14027

**Published:** 2021-03-21

**Authors:** Amr Elmoheen, Benny R Ponappan, Stanley John, Noushad Thayyil, Khalid Bashir

**Affiliations:** 1 Emergency Medicine, Hamad Medical Corporation, Doha, QAT; 2 Emergency Medicine, College of Medicine, Qatar University, Doha, QAT; 3 Medicine, Qatar University, Doha, QAT

**Keywords:** urinoma, uti, ureteral calculi, renal colic

## Abstract

This article describes the case of a 38-year-old male who presented to the ED with three days history of gradually progressing right-sided lower abdominal pain, which had increased in severity two hours prior to his ED visit. The patient was anorexic but denied experiencing any fever, urinary malfunctions, or chills. Blood tests showed an elevated serum creatinine level of 123 umol/L and a high C-reactive protein level of 62 mg/L. Bedside point-of-care ultrasound (POCUS) imaging showed right-sided mild hydroureteronephrosis with surrounding perinephric fluid. Initially, based on the patient’s presentation and clinical findings, appendicitis or ureteric colic was strongly suspected. CT of the abdomen with contrast revealed urinoma measuring 16 cm, and there was a 3.2 mm calculus in the distal part of the right ureter, with perinephric and periureteric fat stranding.

This rare phenomenon requires prompt care. Delayed medical treatment may result in complications like hydronephrosis, abscess, distorted electrolyte levels, and gradual loss of renal function. Small urinomas are usually treated conservatively, while large-sized urinomas often require aggressive medical treatment. A drainage catheter under CT or ultrasound guidance may be done, and additional decompression and drainage may be needed with percutaneous nephrostomy tubes. The fluid and urine culture guide antibiotic treatment.

## Introduction

Urinomas and urinary leaks are rare conditions, almost less than 1% of renal injuries [1]. They are caused by injury to the urinary collecting system [2]. Urinomas are mainly characterized by urine collections in the retroperitoneum, especially in the perirenal space, usually due to leakage of the urinary tract as a result of trauma, post-instrumentation, or obstruction. Extravasation of urine into the retroperitoneal space can trigger a local inflammatory response on the perirenal fat [2]. This may result in lipolysis and urinoma (urine encapsulation). Urinoma as the first manifestation of obstructive uropathy is uncommon. We report a case of obstructive distal ureteric stone who first presented in the ED with urinoma formation.

## Case presentation

A 38-year-old gentleman presented to the ED with three days history of gradually progressing right-sided lower abdominal pain, which had increased in severity two hours before his ED visit. According to the patient, the condition began while he was at home. The patient denied experiencing any traumatic event. The patient had associated anorexia but no fevers, chills, or urinary disorders. He had no significant past medical illness and no previous or recent surgical interventions.

Physical examination of the patient showed an appearance of wellness and anxiety. He was vitally stable with a temperature of 36.5°C, a pulse of 95 beats per minute, a respiratory rate of 19 breaths per minute, and O2 saturation of 99% on room air.

He was fully conscious and had no neurological deficit. His cardiovascular and respiratory system examination was normal. His abdomen examination revealed right iliac region tenderness with no rebound tenderness. His scrotal examination did not show any abnormalities.

**Figure 1:**
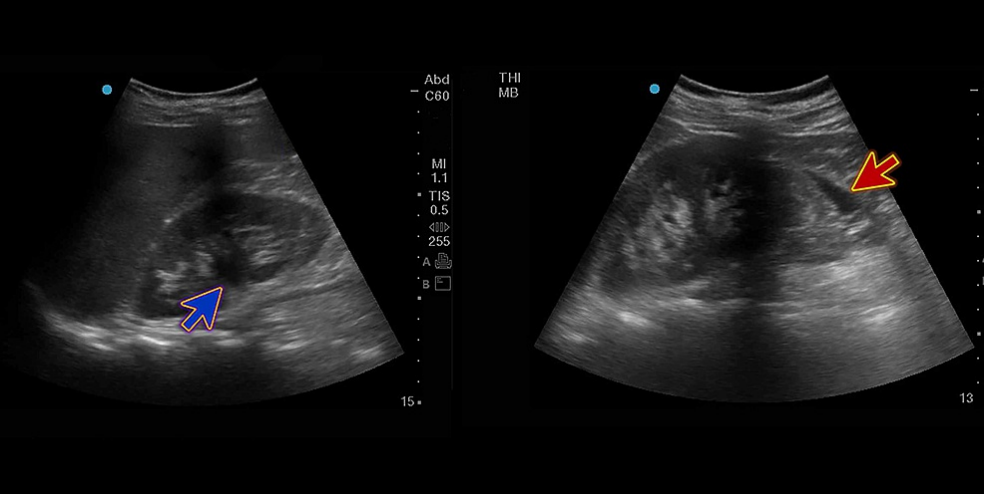
Bedside POCUS showing right-sided mild hydroureteronephrosis (blue arrow) with surrounding perinephric fluid (red arrow). POCUS, point-of-care ultrasound

The patient’s blood tests revealed mild leukocytosis of 11.6 x 1000/uL, hemoglobin of 14.3 g/dL, platelets of 219 x 1000/uL, blood urea of 6.0 mmol/L, elevated serum creatinine levels of 123 umol/L, and a high C-reactive protein level of 62 mg/L. The urine dipstick exam was normal. Other lab parameters were unremarkable. Bedside point-of-care ultrasound (POCUS) imaging showed right-sided mild hydroureteronephrosis with surrounding perinephric fluid (Figure 1).

**Figure 2:**
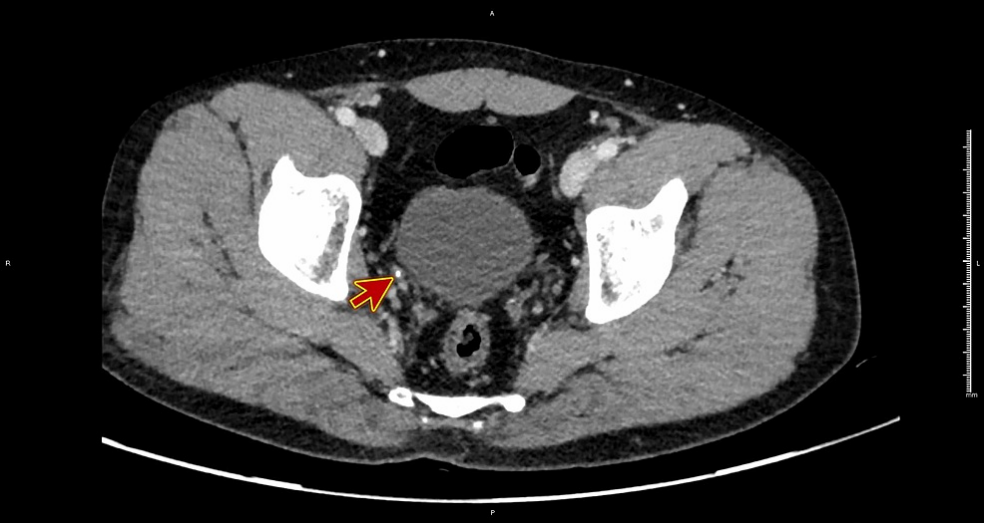
CT scan of the abdomen and pelvis (plain) showing a 3.2 mm calculus in the distal part of the right ureter (red arrow).

**Figure 3:**
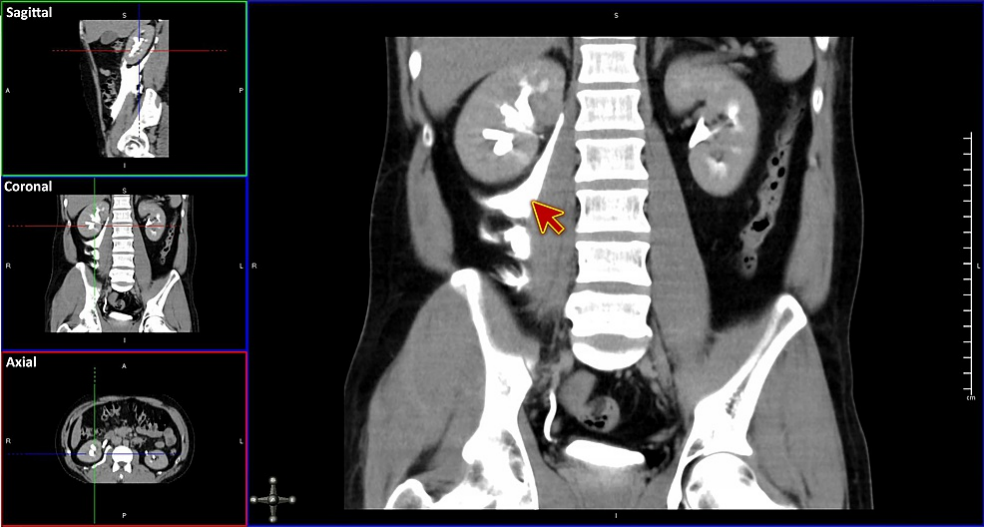
CT scan of the abdomen and pelvis with IV contrast showing perinephric and periureteric fat stranding with contrast extravasation (red arrow), going with urinoma. Multiplanar reformation or reconstruction (MPR) showing Sagittal, Coronal, and Axial planes

A CT scan of the abdomen and pelvis was obtained using IV contrasted dye for confirmation and assessing a suspected perinephric collection. There was a 3.2 mm calculus in the distal part of the right ureter, with perinephric and periureteric fat stranding. Also, there was retroperitoneal fluid, extending from posterior perinephric space, inferiorly into the pelvis, approximately measuring 1.8 cm x 5.2 cm x 16 cm with contrast extravasation, going with urinoma, and the possible site of perforation is the pelvicalyceal system towards calyx or in the upper ureter (Figures 2-3).

Differential diagnosis was based on the patient’s presentation and clinical findings. We strongly suspected appendicitis or ureteric colic. CT of the abdomen with contrast revealed urinoma measuring 16 cm.

The patient was treated with IV fluids and analgesics. Urology was consulted, and the patient was admitted under their care. Urology decided to relieve the obstruction and manage the urinoma conservatively as it was small at presentation and will reabsorb without requiring any surgical operation. The patient underwent cystoscopy and retrograde pyelography, followed by a double-J stent insertion. There were no immediate complications from the procedure.

**Figure 4:**
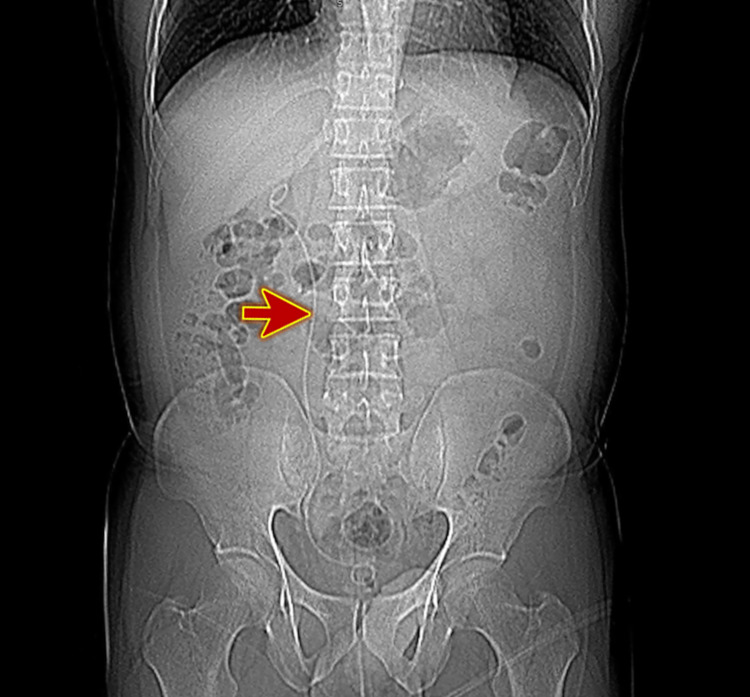
KUB X-ray (scout view) showing the stent in position (red arrow). KUB, kidney, ureter, and bladder

A kidney, ureter, and bladder (KUB) X-ray was done postoperatively, which confirmed the stent position (Figure 4).

The patient was discharged the next day with follow-up in a post-operative urology clinic for removal of the stent. The stent was removed after 20 days without complications, and the patient regularly follows with the out-patient clinic. 

## Discussion

Urinomas may be unilateral or bilateral. The patient may experience symptoms that manifest as encapsulated or confined collection [3]. Urinomas, which are encapsulated collections of extravasated urine, are of two types: subcapsular urinoma and perirenal urinoma. Initially, researchers thought that urinomas protected renal function. However, recent research questions the protective effect of urinomas, as some patients have experienced impaired renal function in the kidney that lies on the same side (ipsilateral) as the urinoma [4-5]. A study by Patil et al. showed that there was no significant difference in the renal function of urinoma patients with ascites compared to those without urinoma [4]. And as verified by Wells et al., urinoma has a characteristic pop-off function that has a long-term protective effect on renal function [6].

The underlying pathology of urinomas may be obstructive or nonobstructive. Obstructive causes of urinomas include ureteral calculi, pregnancy, prostate enlargement, pelvic masses, post-radiation scarring, retroperitoneal fibrosis, congenital anomalies, enlargement of lymphatic glands, and posterior urethral valves. Nonobstructive causes include injuries due to genitourinary surgery, retroperitoneal surgery, gynecological surgery or pelvic surgery, and trauma to the kidneys [7].

Without a doubt, urinoma is a rare disorder [1]. Patients may present with or without symptoms, ranging from an acute abdomen to pain and vague malaise. Injuries may be indicated by changes in urine output or hematuria [2]. Trauma to the urinary tract is the major cause of urinoma in adults. Ureteral calculi and other obstructive causes are less likely to manifest as urinoma. Urinoma is formed via a mechanism known as pyelosinus backflow of urine, which can occur when intrapelvic pressures increase above 35 cm H2O, followed by caliceal fornice rupture.

Urinomas arising from ureteral calculi are a rare occurrence. Calculi increase intrapelvic pressures, backflow of pyelosinus, and caliceal fornice rupture, which leads to extravasation of urine [7]. In a subcapsular urinoma, there is urine between the capsule and parenchyma of the kidney. Conversely, perirenal urinomas are characterized by the collection of urine in between the capsule and the Gerota’s fascia [8]. In many cases, there is leakage of urine into the perirenal or subcapsular space within Gerota’s fascia with wide extravasation, along with the flow of urine across through lymphatic vessels [9]. In the event that it extends inferiorly, it will move along the iliopsoas region right beneath the inguinal ligament to tissues of the buttocks, thighs, scrotum, or peritoneum.

Most often, urinomas are small at presentation and will reabsorb without requiring any intervention. In the event of a fever, a significant injury, urosepsis, or a large expanding urinoma that can be compressing, a urological procedure is often required to relieve the urinoma [2]. A delay may cause the formation of an abscess, hydronephrosis, electrolyte imbalance, and urosepsis. Fluid analysis usually shows a high level of creatinine and low glucose concentration relative to serum. Pyuria and hematuria are observed in urinalysis [4]. The patient may be evaluated via renal ultrasonography and then with a CT KUB without contrast involving a low-dose noncontrast phase alongside a delayed image phase done 10 minutes after administering IV contrast for confirmation and assessing a suspected perinephric collection [10-11]. CT is considered the gold standard because it highlights the relationship between the kidney and the urinoma, the fascial planes, and the ureter [12]. First-line intervention is usually the placement of a catheter into the urinoma, along with the administration of empiric antibiotics. If the catheter cannot drain the urinoma as it should, the clinician may then place a percutaneous nephrostomy tube to enhance drainage. Insertion of a ureteral stent promotes healing [13-14]. Surgery is carried out in severe cases. Aggressive measures are best avoided by early diagnosis and prompt treatment.

## Conclusions

Urinomas, are, without a doubt, a rare occurrence. The underlying pathology of urinomas may be obstructive or nonobstructive. Diagnosis can be made via a dedicated CT scan. Conservative treatments, along with drainage techniques, may help to prevent further complications.
